# Joint Mobility Protection during the Developmental Age among Free Climbing Practitioners: A Pilot Study

**DOI:** 10.3390/jfmk5010014

**Published:** 2020-02-17

**Authors:** Ludovica Gasbarro, Elvira Padua, Virginia Tancredi, Giuseppe Annino, Michela Montorsi, Grazia Maugeri, Agata Grazia D’Amico

**Affiliations:** 1 Department of Human Sciences and Promotion of the Quality of Life, San Raffaele Roma Open University, 00166 Rome, Italy; gasbarroludovica@gmail.com (L.G.); elvira.padua@uniroma5.it (E.P.); agata.damico@uniroma5.it (A.G.D.); 2 Department of Systems Medicine, University of Rome Tor Vergata, 00166 Rome, Italy; virginia.tancredi@uniroma5.it (V.T.); g_annino@hotmail.com (G.A.); 3 Section of Anatomy, Histology and Movement Sciences, Department of Biomedical and Biotechnological Sciences, University of Catania, 95123 Catania, Italy

**Keywords:** joint mobility, development phases, sport climbing, stretching

## Abstract

Sport-climbing popularity increased intensely over the past years. Particularly, children’s and adolescents’ interest therein is constantly growing. Despite a large effort in preventing injuries and muscle overloads, a fine-tuned training for each sensitive phase of child development is still needed. The objective of the study was to evaluate an innovative training program aimed at the preservation of joint mobility during the developmental age. This article relies on the results of a steady training program allowing to retain joints integrity among the practice of sport climbing in children. Joint mobility changes have been monitored before and after a one-year training program in fifteen subjects aged between 8 and 18 years. Subjects were divided into three groups depending on age (Turgor Secundus, Proceritas Secunda and Turgor Tertius). The motor tests administered were the sit-and-reach test, coxo-femoral mobility test and scapula–humeral mobility test. Our results showed that one-year training improved joint mobility at each analyzed phase, suggesting that this training program could improve mobility and flexibility. Given the importance of joint mobility preservation for discipline-related injuries prevention and eventually recovering, it is essential to provide a specific training program as a route to approach sport climbing, and even more importantly, at an early age. This work represents a preliminary study in order to demonstrate both efficacy on the joint mobility and the requirement of our playful work to support the global sport-climbing workout.

## 1. Introduction

In the last decades, rock climbing has become a popular sport among adults, adolescents and children, also since it being included in the 2018 Summer Olympic Youth Games in Buenos Aires and in the Summer Olympic program of the 2020 Games in Tokyo [1,2]. In using only their bare hands and climbing shoes to perform a range of hand and foot holds, athletes climb vertical walls in three disciplines: Speed, with two climbers simultaneously climbing a route on a 15 m wall; Bouldering, with athletes performing in a given time a number of fixed routes on a 4m wall; and Lead, with athletes climbing in a given time a 15 m wall [3]. It is possible to practice it not only outdoor, as now there also are indoor structures; so, this sport is becoming widespread in adults and adolescents, too.

Rock climbing has several benefits on both physical fitness and mental care. In fact, this sport improves strength and endurance [4,5]. A recent study [6] has demonstrated that rock climbing significantly improves muscle power, ability to produce a maximal force in a short time, muscle endurance and skills to perform a continuous muscle work for a long time. This kind of sport also has beneficial effects on depressive disorders due to the positive effect on cognitive control of the physical activity connected to high levels of coordination [7]. It has been demonstrated that only a single rock-climbing session may have a positive effect in major depressive disorder [8].

Due to the increased popularity, the average age of rock climbers is decreasing, and more and more young athletes win medals. Unfortunately, the increased number of young rock climbers involves a major risk of an injury in the developmental age [9].

During climbing very small parts of the hands and feet are in contact with the climbing surface [10,11,12] and climbers have to support and/or lift their bodies by combining a variety of finger grips with balanced, complex vertical and lateral movements and position holds [13,14]. To guarantee grip and stability, specialized smooth climbing footwear with a sticky rubber sole are used. Although climbing shoes should fit snug without pain, the climbers tend to use smaller sizes, which could cause injuries or foot deformation [15]. 

Physical activity is essential to maintain good health and guarantee a better quality of life. The major sport-related benefits involve not only the body but also the mind. In fact, physical activity improves motor and cognitive skills, reduces risk for obesity, exerts positive effects on blood pressure and lipidemia, as well as decreasing the risk of depression and other mental disorders [16,17]. However, incorrect or excessive training may lead to adverse effects, including musculoskeletal injuries, recently described in young soccer players, as well as social–occupational dysfunction [18,19]. In particular, in rock climbing the articulations are very solicited and the scientific literature reports injuries in fingers or in shoulders [15,20]. In particular, Garcia et al. [21] examined hands and fingers of young climbers versus a control group of non-climbers and they showed a non-physiological development of fingers. Similarly, another study [22] showed differences in the static scapular position between rock climbers and a control group.

In particularly the developmental age there is a major risk of sprains, strains and fractures with chronicle injuries at the upper extremity and acute injuries at the lower extremity [23]. A survey among rock climbers [24] reports 90% upper extremity injuries, and among these the most common are fingers followed by shoulder/arm and elbow/forearm.

Rock climbing is positively related to increased bone mineral content, weight and mass body [15]. However, for climbers a low body mass index is an ideal anthropometric requirement. Therefore, they perform a restricted diet to maintain a low body weight, by inducing a negative effect on their health and in particular on their bones [25,26]. Moreover, the stresses associated with rock climbing may have the potential to create scapula–humeral or coxo–femoral injuries [22].

Despite the increase in rock-climbing practices and the consequent increase in studies on the benefits or injuries of climbing, little data are available on adolescents.

To envision the development of effective preventative measures for preserving the joint mobility and health of youth practitioners, the aim of this preliminary study was to evaluate the effects of a specific pre-training rock climbing program to be administered to athletes according to their developmental status. 

Therefore, in this preliminary study we evaluated an innovative training program to preserve joint mobility during the developmental age. Children were subjected to a steady training program in order to retain joint integrity and to avoid climbing-associated injuries.

## 2. Materials and Methods 

### 2.1. Subjects

Fifteen subjects aged between 8 and 18 years, working out regularly in sport climbing and joint mobility, were divided into 3 groups consisting of 5 athletes each according to their ages. Turgor Secundus (3 boys and 2 girls; aged between 8 and 10), Proceritas Secunda (2 boys and 3 girls; aged between 12 and 14) and Turgor Tertius (2 boys and 3 girls; aged between 15 and 18). 

Table 1 reports the anthropometric characteristics of the athletes, divided in subgroups according to their developmental phase.

This study received the consence by the Institutional Research Board of the University San Raffaele of Rome, Italy and all subjects’ legal tutors gave written informed consent in respecting the ethical principles of the Declaration of Helsinki. All participants were novices regarding climbing experience.

### 2.2. Methodology

Knowledge of the main physiological changes and the sensitive phases of development is fundamental for the educator, as on the basis of this information he can develop a program suitable for the child’s needs. The sensitive phases of development are periods of growth in which the child will be predisposed to increase some motor skills rather than others. Furthermore, the differential growing of the bones, nervous system and muscles in the different evolutionary phases must be taken into consideration in the programming to avoid injuries and the onset of paramorphism.

Our study was conducted for 12 months, and during this experimental period the climbing training program was carried out according to the coach’s training plan. Children worked out twice a week for 90 min divided as follow: 15 min for stretching, 20 min for specific training to improve joint mobility and 55 min for climbing. 

Joint mobility variation was assessed over a one-year training period. All the measurements were performed at baseline (time 0; start measurement) and repeated at the end of the training period (time 1; after 12 months) in order to evaluate scapula–humeral and coxo–femoral joint mobility and spine flexibility. The measures recorded in time 0 were considered our control data and the measures obtained at the end of training program were compared to data recorded in time 0 in order to calculate the improvement of joint mobility for each group. 

Tests have been performed as follows:

Sit-and-reach test: Participants sat on the floor with legs extended, backs straight and feet resting on a cube with a graduated wooden board above it. The participants are asked to slide their hands above the wooden board, keeping the knee extended [27].

Hip joint mobility: Participants are sitting with their back against the wall, slowly reaching the maximum opening of their hips. The measurement is then taken between the inner ankles [28]. 

Scapulo–humeral mobility test (measurement of shoulder mobility): Participants are in an upright position, holding a stick, bringing the arms outstretched behind the trunk and reaching the starting position without bending the arms. Then, the minimum distance between the hands while the subject is holding the stick was measured [29].

### 2.3. Training Regime 

Participants of the three experimental groups underwent a specific training program 2 times/week, performing 15 min of general warm-up before training. The specific training program for each phase was done for 20 min before rock climbing.

According to each phase, the following training methods were proposed in order to improve joint mobility.

**Figure 1:**
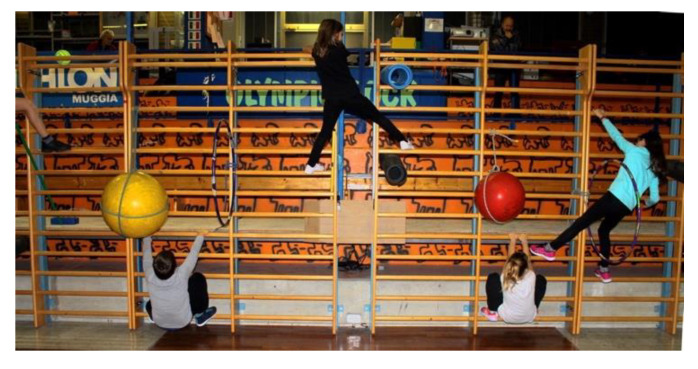
Children performing exercises starting from the left side of the wall bar and moving sideways to pass over.

The Turgor Secundus phase: Obstacle course on wall bars (Figure 1) to preserve a good degree of flexibility and to practice the typical positions of sport climbing:

Exercise 1: The aim was to start from the left side of the wall bar and moving sideways to pass over, under and through some obstacles, following a predetermined path, without falling (Figure 1). 

**Figure 2:**
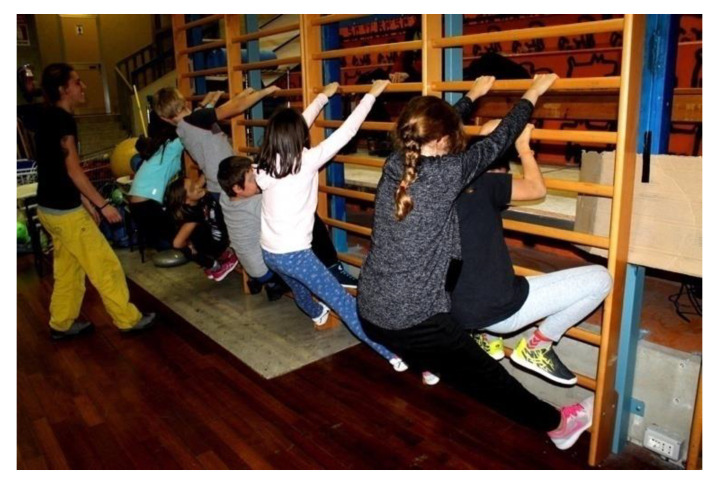
Overview of spider and crab positions.

Exercise 2: The game of spiders and crabs: half of the children have to positioning themselves in a line, keeping their arms and legs outstretched, forming a large arch. The crabs will have to come through it (Figure 2).

**Figure 3:**
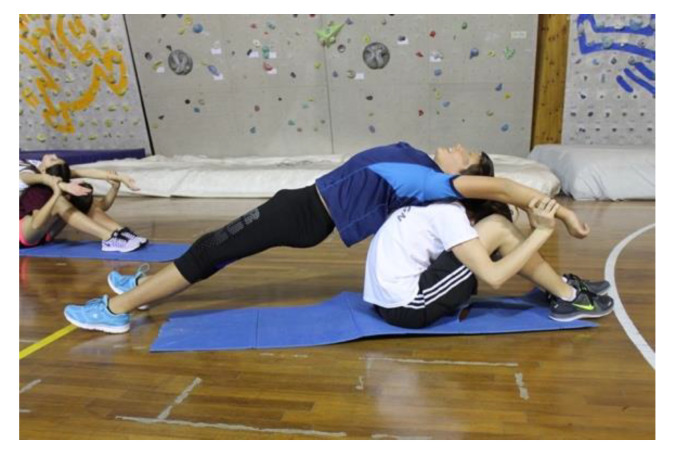
Final position of the spine mobilization exercise.

**Figure 4:**
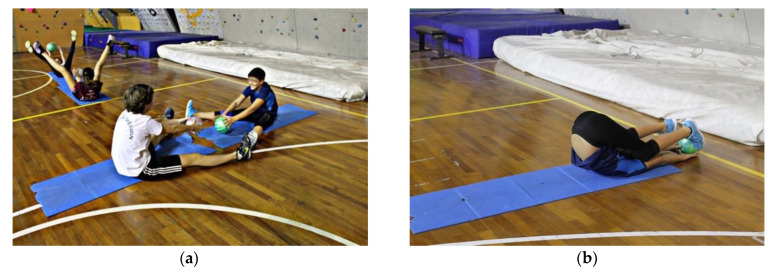
(**a**) Starting position of the mobilization of the spine and coxo–femoral joint exercises. (**b**) Ending position of the mobilization of the spine and coxo-femoral joint exercises.

**Figure 5:**
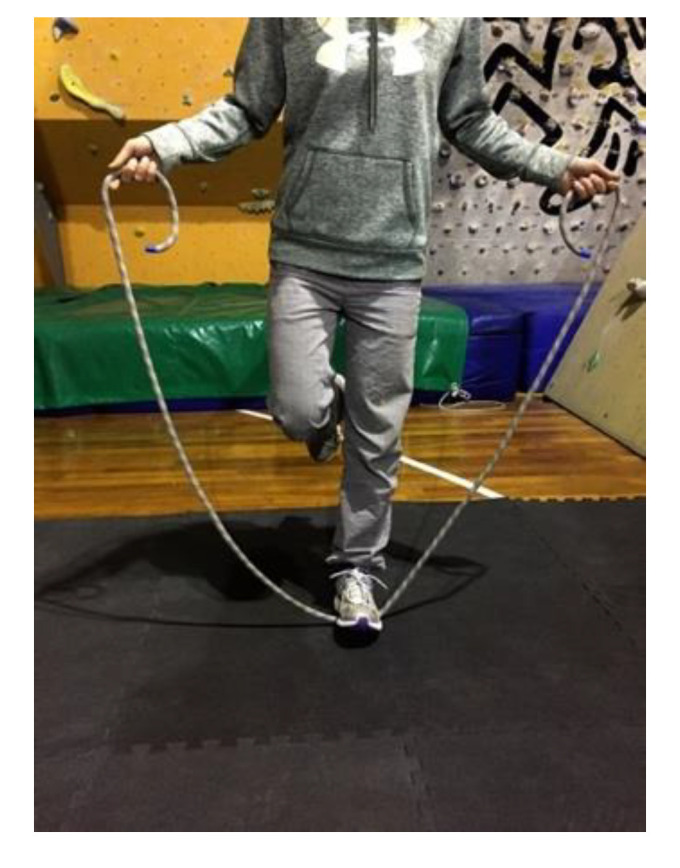
Flex foot and rope locked by the foot.

The Proceritas Secunda phase: Mobilization of the spine (Figure 3 and Figure 4a,b) and the tibio–tarsic joint (Figure 5):

Exercise 1: Hamstring stretch involving two subjects (A and B) that have to sit back-to-back on the floor with their legs extended forward. Subject A is stretching back by abducting his/her arms upwards slightly pressing its weight, and simultaneously subject B reaches for his/her toes. The position has to be kept constant for 4–5 s, after which this exercise is repeated but with the subjects changing their roles (Figure 3).

Exercise 2: The subjects sit up tall in the straddle position feet-to-feet, with straightened legs, and holding a ball in their hands (Figure 4a). They slowly lean down on the back, closing their legs and curling the pelvis inward until their toes touch the ball behind their heads, and then back to the starting position (Figure 4b).

Exercise 3: In single foot support, rotate the rope forward without jumping. After rotating the rope forward, to pass the rope beyond the foot, flex the foot and then extend it. Do the same with the other foot (Figure 5).

**Figure 6:**
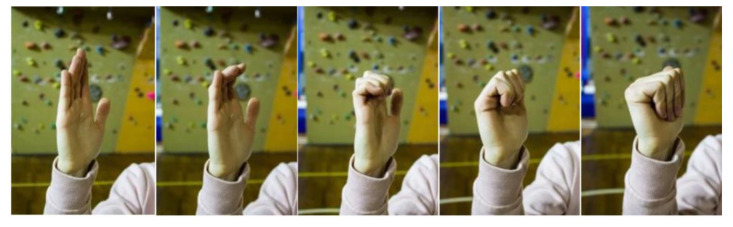
Mobility exercises for fingers from starting to ending positions.

**Figure 7:**
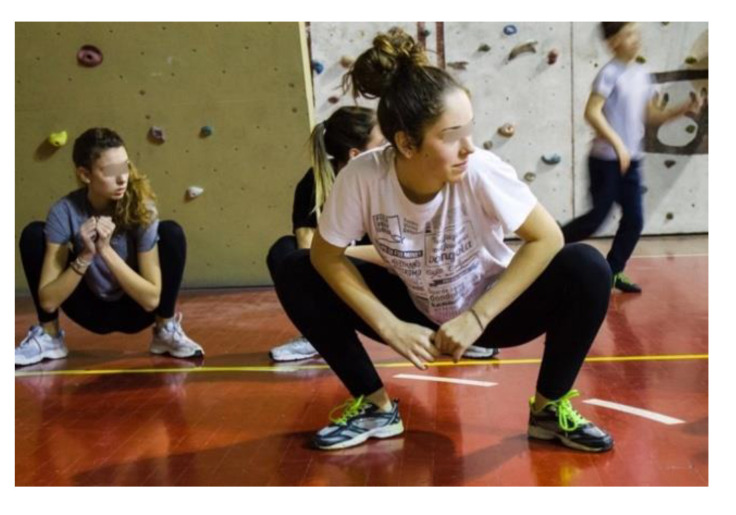
Squat position for mobilization of the coxo–femoral joint.

**Figure 8:**
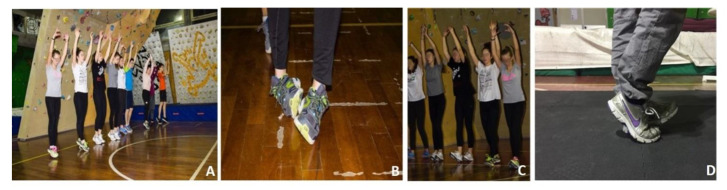
(**A**) Toe walking with raised arms; (**B**) foot rolling forward; (**C**) heel walking with raised arms; (**D**) feet supination and pronation walking forward.

Turgor Tertius phase: Mobility exercises for the fingers (Figure 6), coxo–femoral (Figure 7) and tibio–tarsic joints (Figure 8): 

Exercise 1: Starting with a hand fully opened; perform four closing finger movements, through which both the metacarpophalangeal and the interphalangeal joints will be stimulated (Figure 6).

Exercise 2: The subject is crouched down, the feet are in a wide stance with the toes turned out, the back outstretched, the heels on the ground and the elbows slightly pushing the knees outwards. Then, small rotations of the ankles are made first in one direction and then in the other (Figure 7).

Exercise 3: Four different types of gaits are proposed. First, toe walking with raised arms. Second, heel walking with raised arms. Third, foot rolling forward. Lastly, feet supination and pronation walking forward (Figure 8A–D).

### 2.4. Statistical Analysis

Data collected at baseline (pre-training) and after (post-training) the one-year experimental period are presented as the mean ± SD. The assumption of normality was verified by means of the Kolmogorov–Smirnov test. Then, for each subgroup a paired *t*-student test was used to ascertain differences between pre-training and post-training data. Throughout the study, the level of significance was set at *p* ≤ 0.05. Statistical analysis was conducted using GraphPad Prism version 6.

## 3. Results

Athletes have carried out the proposed activities for the entire duration of the study over and above the regular workout, with the exception of a female belonging to the group Proceritas Secunda who interrupted the training program for one and a half months. However, data from subjects who dropped out were used for preliminary comparisons. All groups considered in our study have shown an improvement in joint mobility (Table 2). However, differences in training response among the considered phases have been identified. 

**Figure 9:**
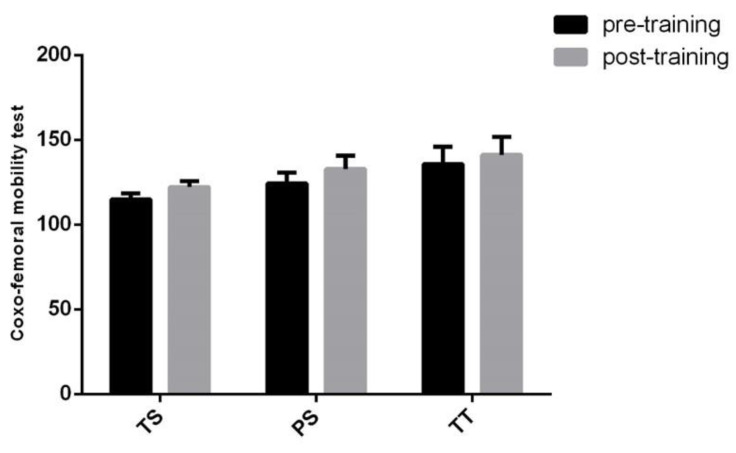
Coxo–femoral mobility test. The results are represented in bar graphs as the mean ± SD of the different phases considered (Turgor Secundus, Proceritas Secunda and Turgor Tertius).

**Figure 10:**
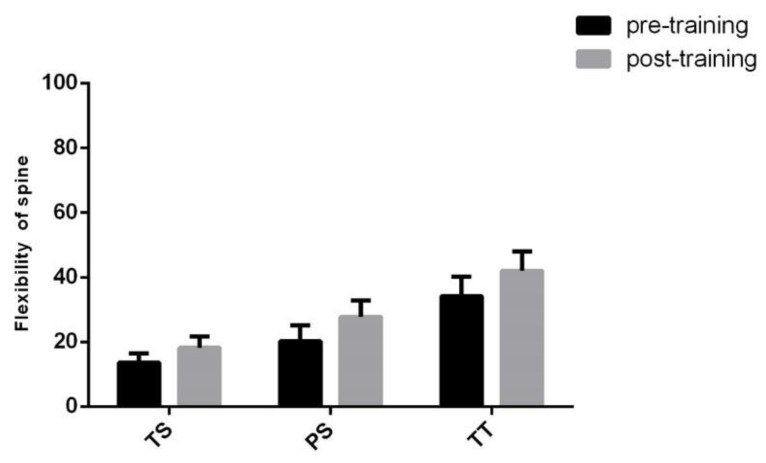
Flexibility of the spine. The results are represented in bar graphs as the mean ± SD of the different phases considered (Turgor Secundus, Proceritas Secunda and Turgor Tertius).

**Figure 11:**
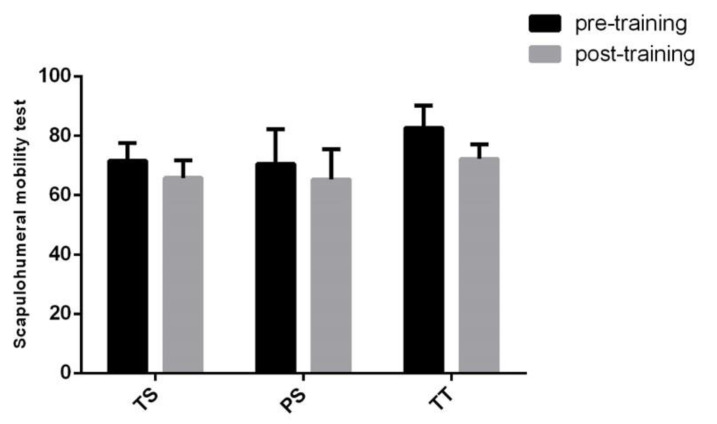
Scapulo–humeral mobility test. The results are represented in bar graphs as the mean ± SD of the different phases considered (Turgor Secundus, Proceritas Secunda and Turgor Tertius).

Proceritas Secunda showed a positive response in coxo–femoral mobility and lower vertebral column flexibility 12 months after the start of the training program (Figure 9 and Figure 10), whereas scapula–humeral mobility did not report benefits after joint-specific training (Figure 11). On the other hand, scapula–humeral mobility remarkably improves in the Turgor Tertius phase when compared to both Turgor Secundus and Proceritas Secunda (Figure 11). Indeed, this is consistent with the normal development of the shoulder joint that tends to complement the most advanced evolutionary phases. However, this does not apply to the other two phases (Figure 11). The Turgor Tertius group have also recorded an improvement in spine flexibility as reported in Figure 10.

**Figure 12:**
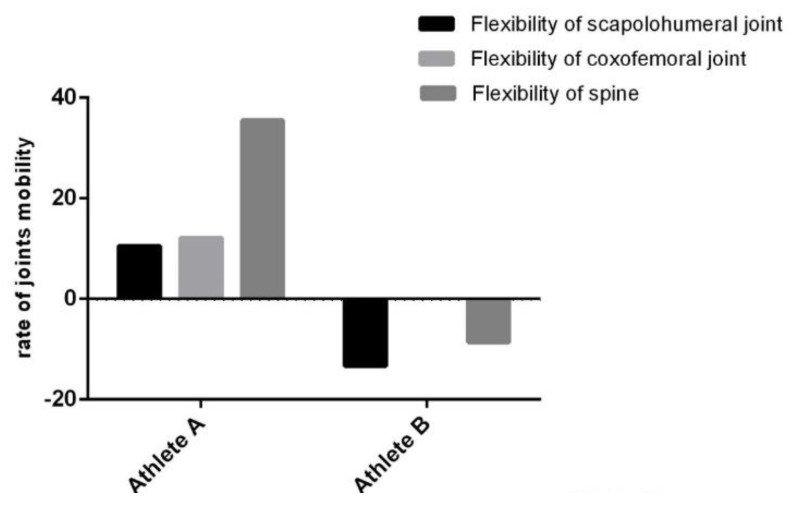
Comparison between the percentage mean variations of two athletes: Athlete A has completed the training, while Athlete B has totally interrupted the workout for one and a half months.

Regarding Proceritas Secunda, we further compared two 14-year-old subjects (Figure 12). Athlete A has completed his/her training, whereas Athlete B has totally interrupted the workout for one and a half months. The latter has been previously excluded from the overall analysis. It is noteworthy that we clearly identified a remarkable impairment in terms of mobility and flexibility as a result of the training discontinuation of Athlete B. 

## 4. Discussion

This work represents a preliminary study in order to demonstrate both the efficacy on joint mobility and the requirement of our playful work, to support the global sport-climbing workout. Indeed, the increased interest in rock climbing practices creates the need to know all the possible sport-related injuries in order to prevent and treat them. Hence, the importance to study the main joints involved in climbing and that are at risk of injuries, such as joints of the hand and fingers, shoulders, the hip and ankles, in order to develop a preparation workout suitable for the discipline.

Since sport climbing does not need specific anthropometrical characteristic, and for this reason it can be practiced by everyone [30], a specific training program in this sport can improve one’s strength and resistance; essential skills that would allow to carry out increasingly difficult passages. In fact, climbers perform movements that require an isometric effort, resulting in an enhanced muscle tone to obtain a strong and harmonious body. Moreover, although it is not an aerobic discipline, it develops a good cardiovascular training; this is also due to the adrenaline that develops while climbing [31]. 

Because of a child’s incomplete maturation of both bone and muscular structures, and the laxity of ligaments, early and specific training would reasonably provide extensive room for improvement. We have proposed a playful training workout as a mobility workout in the first sensitive stages.

In the present work, we have conducted a preliminary study in an attempt to prove that a specific training program is fundamental for mobility and flexibility improvement. Participants enrolled for our study were novices regarding climbing experience and they worked out twice a week for 90 min in agreement with the coach’s training plan for 12 months.

Although the reported data did not reach statistical significance, we have recorded an improvement in joint mobility after the playful training program. However, not all the recorded ameliorations were statistically significant. This might be due to external factors affecting joint mobility, such as anatomical and physiological differences among subjects. However, this can be also ascribable to the low sample size. The latter stems from the direct correlation between the standard deviation value and the sample size.

Moreover, by comparing continuous and discontinuous training between two athletes, we have suggested the importance of early and constant training (Figure 12). Indeed, the suspension of a joint-specific workout can lead to a blockage or even a regression of the subject’s abilities. Thus, injuries require prompt treatment in order to avoid transient and permanent physical impairment. 

Even if the study was conducted on a low number of subjects, this represent a starting project in order to develop a specific training program for each developmental phase.

## 5. Conclusions

The recent proliferation of indoor climbing gyms and well-protected sport climbing areas have made sport climbing accessible to everyone. Neither an age group nor a pre-existing medical condition serves as a contraindication for sport climbing in the first instance. Sport climbing is an engaging mental and physical activity that contributes to an increase in muscle mass and strength, dynamic balance, and other health benefits. However, in line with any exercise prescription, guidelines for sport-specific participation are desirable. Taking into account what was shown in this preliminary study, early and constant joint-mobility training that start from the early stages of the developmental age is fundamental.

In conclusion, we suggest that joint-mobility exercises from the early stages of development will allow children to move harmoniously, even on the climbing walls.

## Figures and Tables

**Table 1:** Characteristics of participants recruited to conduct the present study expressed as mean fold change ± SEM.

Characteristic	TURGOR SECUNDUS (Mean ± SEM)	PROCERITAS SECUNDA (Mean ± SEM)	TURGOR TERTIUS (Mean ± SEM)
Age	8.80 ± 0.37	13.00 ± 0.45	16.60 ± 0.51
Weight	28.80 ± 1.46	49.00 ± 2.97	55.20 ± 3.61
Height	128.60 ± 1.81	160.00 ± 2.98	167.80 ± 3.44
BMI	17.46 ± 1.03	19.15 ± 1.08	19.53 ± 0.73

**Table 2:** Percentage of improvement in flexibility in the different phases expressed as mean fold change ± SD.

PHASE	SCAPULO–HUMERAL JOINT (Mean ± SD)	COXO–FEMORAL JOINT (Mean ± SD)	SPINE (Mean ± SD)
TURGOR SECUNDIS	8.19 ± 1.76	6.46 ± 0.74	35.95 ± 3.16
PROCERITAS SECUNDA	7.82 ± 1.08	8.58 ± 2.07	36.28 ± 4.15
TURGOR TERTIUS	11.85 ± 2.20	4.20 ± 1.18	27.97 ± 7.71

## References

[1] https://www.ifsc-climbing.org/index.php.

[2] https://tokyo2020.org/en/.

[3] https://www.ifsc-climbing.org/.

[4] Michailov M.L., Baláš J., Tanev S.K., Andonov H.S., Kodejška J., Brown L. (2018). Reliability and Validity of Finger Strength and Endurance Measurements in Rock Climbing. Res. Q. Exerc. Sport.

[5] Baláš J., Pecha O., Martin A.J., Cochrane D. (2012). Hand–arm strength and endurance as predictors of climbing performance. Eur. J. Sport Sci..

[6] Li L., Ru A., Liao T., Zou S., Niu X.H., Wang Y.T. (2018). Effects of Rock Climbing Exercise on Physical Fitness among College Students: A Review Article and Meta-analysis. Iran. J. Public Health.

[7] Voelcker-Rehage C., Godde B., Staudinger U.M. (2011). Cardiovascular and Coordination Training Differentially Improve Cognitive Performance and Neural Processing in Older Adults. Front. Hum. Neurosci..

[8] Kleinstäuber M., Reuter M., Doll N., Fallgatter A.J. (2017). Rock climbing and acute emotion regulation in patients with major depressive disorder in the context of a psychological inpatient treatment: A controlled pilot trial. Psychol. Res. Behav. Manag..

[9] Lutter C., El-Sheikh Y., Schöffl I., Schöffl V. (2017). Sport climbing: Medical considerations for this new Olympic discipline. Br. J. Sports Med..

[10] Quaine F., Vigouroux L., Martin L. (2003). Effect of simulated rock climbing finger postures on force sharing among the fingers. Clin. Biomech.

[11] Roloff I., Schöffl V.R., Vigouroux L., Quaine F. (2006). Biomechanical model for the determination of the forces acting on the finger pulley system. J. Biomech..

[12] Vigouroux L., Quaine F. (2006). Fingertip force and electromyography of finger flexor muscles during a prolonged intermittent exercise in elite climbers and sedentary individuals. J. Sports Sci..

[13] Schöffl V., Einwag F., Strecker W., Schöffl I. (2006). Strength measurement after conservatively treated pulley ruptures in climbers. Med. Sci. Sports Exerc..

[14] Schöffl I., Einwag F., Strecker W., Hennig F., Schöffl V. (2007). Impact of taping after finger flexor tendon pulley ruptures in rock climbers. J. Appl. Biomech..

[15] Morrison A.B., Schöffl V.R. (2007). Physiological responses to rock climbing in young climbers. Br. J. Sports Med..

[16] Timmons B.W., Leblanc A.G., Carson V., Connor Gorber S., Dillman C., Janssen I., Kho M.E., Spence J.C., Stearns J.A., Tremblay M.S. (2012). Systematic review of physical activity and health in the early years (aged 0–4 years). Appl. Physiol. Nutr. Metab..

[17] Janssen I., Roberts K.C., Thompson W. (2017). Is adherence to the Canadian 24-Hour Movement Behaviour Guidelines for Children and Youth associated with improved indicators of physical, mental, and social health?. Appl. Physiol. Nutr. Metab..

[18] Malm C., Jakobsson J., Isaksson A. (2019). Physical Activity and Sports—Real Health Benefits: A Review with Insight into the Public Health of Sweden. Sports.

[19] Iwame T., Matsuura T., Suzue N., Iwase J., Uemura H., Sairyo K. (2019). Factors Associated With Knee Pain and Heel Pain in Youth Soccer Players Aged 8 to 12 Years. Orthopaedic J. Sports Med..

[20] Schöffl V., Lutter C., Woollings K., Schöffl I. (2018). Pediatric and adolescent injury in rock climbing. Res. Sport. Med..

[21] Garcia K., Jaramillo D., Rubesova E. (2018). Ultrasound evaluation of stress injuries and physiological adaptations in the fingers of adolescent competitive rock climbers. Pediatr. Radiol..

[22] Roseborrough A., Lebec M. (2007). Differences in static scapular position between rock climbers and a non-rock climber population. N. Am. J. Sports Phys. Ther..

[23] Schöffl V.R., Hoffmann P.M., Imhoff A., Küpper T., Schöffl I., Hochholzer T., Hinterwimmer S. (2018). Long-Term Radiographic Adaptations to Stress of High-Level and Recreational Rock Climbing in Former Adolescent Athletes: An 11-Year Prospective Longitudinal Study. Orthop. J. Sport. Med..

[24] Nelson C.E., Rayan G.M., Judd D.I., Ding K., Stoner J.A. (2017). Survey of Hand and Upper Extremity Injuries Among Rock Climbers. Hand.

[25] Turner C.H., Robling A.G. (2004). Exercise as an anabolic stimulus for bone. Curr. Pharm. Des..

[26] Watts P.B. (2004). Physiology of difficult rock climbing. Eur. J. Appl. Physiol..

[27] Anloague P.A., Spees V., Smith J., Herbenick M.A., Rubino L.J. (2012). Glenohumeral range of motion and lower extremity flexibility in collegiate-level baseball players. Sports Health.

[28] Boone D.C., Azen S.P., Lin C.M., Spence C., Baron C., Lee L. (1978). Reliability of goniometric measurements. Phys. Ther..

[29] Sharma J.P. (2011). Tests and Measurements in Physical Education.

[30] Mermier C.M., Janot J.M., Parker D.L., Swan J.G. (2000). Physiological and anthropometric determinants of sport climbing performance. Br. J. Sports Med..

[31] Mermier C.M. (1997). Energy expenditure and physiological responses during indoor rock climbing. Br. J. Sports Med..

